# Metaplastic carcinoma of the breast containing three histological components: a case report

**DOI:** 10.3389/fonc.2024.1470986

**Published:** 2024-12-19

**Authors:** Huan Liu, Gang Zhao, Zhimin Fan, Di Wu, Fengjiang Qu

**Affiliations:** ^1^ Breast Surgery Department, General Surgery Center, The First Hospital of Jilin University, Changchun, China; ^2^ Emergency Surgery Department, The First Hospital of Jilin University, Changchun, China

**Keywords:** breast malignant tumor, squamous cell carcinoma, invasive ductal carcinoma, fibrosarcoma, carcinoma mixed type

## Abstract

Malignant breast tumors mainly arise from the ductal and lobular epithelium, whereas sarcomas, which originate from the stromal tissues of the breast, account for less than 5% of cases. Mostly, these tumors consist of a single tissue type, rendering malignant breast tumors with three distinct tissue types exceedingly rare. We report a unique case of a malignant breast tumor comprising three tissue types: squamous cell carcinoma (approximately 25%), invasive ductal carcinoma (approximately 5%), and fibrosarcoma (approximately 70%). Given the case’s rarity, pre-operative imaging and tumor biopsy failed to yield definitive diagnostic information, we detail the patient’s clinical and therapeutic process, providing insights for physicians on clinical diagnosis and treatment.

## 1 Introduction

Malignant breast tumors are the most common type of cancer in women and are the leading cause of cancer-related deaths among females (1). Based on tissue origin, breast malignancies are classified into epithelial-origin breast carcinomas and mesenchymal-origin breast sarcomas. Breast carcinoma has become the second most prevalent malignant tumor globally, following lung cancer in incidence (2), whereas breast sarcoma is rare (1, 3). The vast majority of malignant breast tumors have a single histopathological component, and cases where both tissue components coexist are exceedingly rare. Here, we report a case of a malignant breast tumor that contains three histological components: squamous cell carcinoma, invasive ductal carcinoma, and high-grade fibrosarcoma.

## 2 Case description

**Figure 1:**
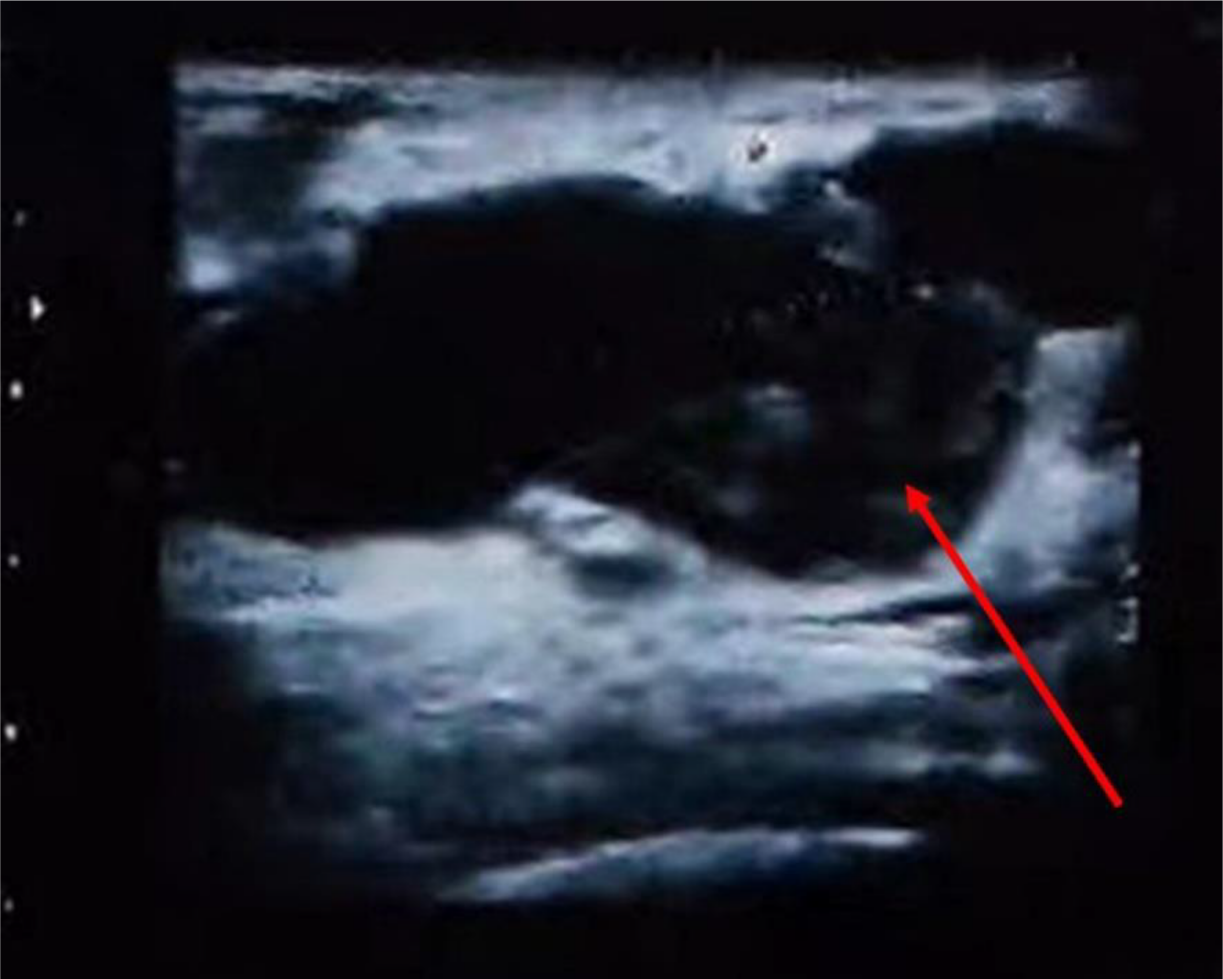
Breast color Doppler ultrasound of the patient at the first admission. The red arrow shows the tumor.

In July 2018, a 68-year-old woman presented to the Breast Surgery Outpatient Clinic at the First Hospital of Jilin University, finding a mass in her left breast discovered five years earlier. Five years earlier, she incidentally found a 1.0cm×1.0cm lump in her left breast, causing occasional pain but it was never formally diagnosed or treated; Two years ago, the lump abruptly grew to 5.0cm×3.0cm, yet it remained untreated; Last month, the skin covering the lump turned red and swollen. She has an 8-year history of hypertension, with no other tumor history or familial predispositions. Physical examination showed redness and swelling in the left breast’s upper outer quadrant, alongside a hard, palpable 6.0cm×6.0cm mass with an irregular surface, unclear boundaries, and limited mobility. Breast ultrasound and mammography identified an irregular, slightly dense mass in the left breast’s upper outer quadrant, measuring 58.2mm×26.1mm and 5.0cm×5.0cm, respectively, both classified as BI-RADS category 3 (see **Figure 1**). Extensive imaging and lab tests, including bone emission computed tomography (ECT) scan, abdominal and Chest computed tomography (CT), neck lymph node, and cardiac ultrasounds, along with complete blood count and liver and kidney function tests, found no significant abnormalities. Upon admission, a biopsy of the left breast mass indicated a complex, fragile tissue composition with atypical cells, suggesting further investigation. Pending exclusion of metaplastic breast carcinoma or fibroepithelial tumor. Immunohistochemical tests show Ki-67(+30%), and P53(+40%), P63(focal+), cytokeratin (CK) 5/6(+), cytokeratin (CK) 7(+), ER (-), pan-cytokeratin (CK-pan) (+), CD68(+), calponin (-), E-cadherin (+), vimentin (+), CD34(-), indicating active cellular proliferation and mutation. Examination of pus and blood cells revealed atypical squamous epithelial cells and numerous lobulated nucleated granulocytes. The final diagnosis was left breast cancer (cT3N0M0) and hypertension.

**Figure 2:**
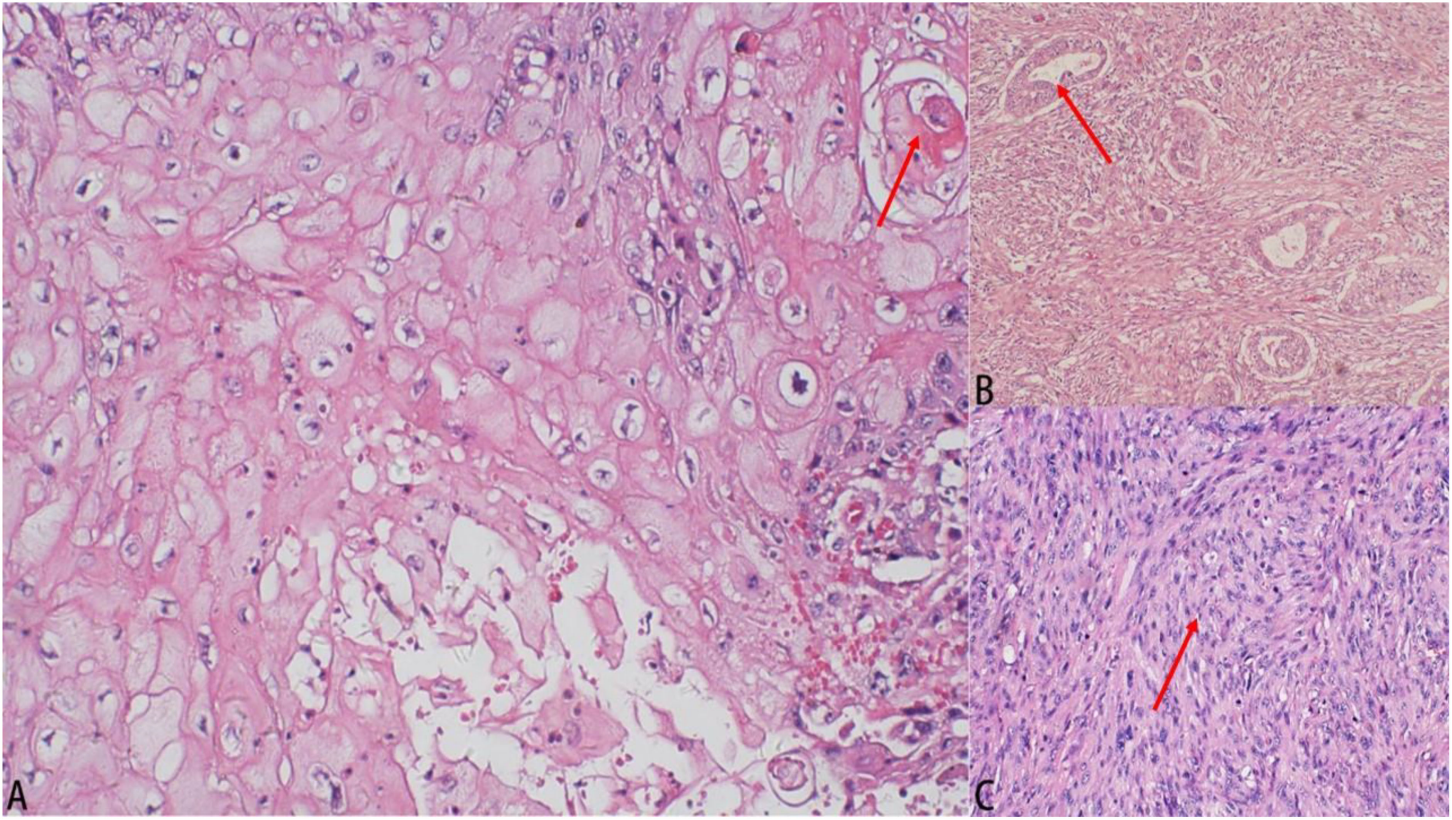
Results of pathological examination. **(A)** Histological image (hematoxylin–eosin staining, 100×): squamous cell carcinoma. **(B)** Histological image (hematoxylin–eosin staining, 100×): invasive ductal carcinoma. **(C)** Histological image (hematoxylin-eosin staining, 100×): High-grade fibrosarcoma.

On August 9, 2018, following preoperative examinations that showed no significant contraindications, the patient underwent a simple mastectomy of the left breast and sentinel lymph node biopsy under general anesthesia. The postoperative pathology report indicated that the tumor was 50% cystic and 50% solid, with the solid portion being slightly papillary, grayish-white, and firm. The total volume of the tumor was approximately 5.0cm×4.0cm×3.0cm. Histologically, it was identified as metaplastic carcinoma/sarcomatoid carcinoma, comprising squamous cell carcinoma (~25%), invasive ductal carcinoma (~5%), and high-grade fibrosarcoma (~70%) (refer to **Figures 2A–C**). The tumor was graded MBNG 3, with no metastasis observed in the sentinel lymph nodes (0/2). Immunohistochemical testing confirmed a mix of squamous cell carcinoma, invasive ductal carcinoma, and sarcoma, showing Ki-67(+30%), ER (-), PR (-), HER2 (invasive ductal carcinoma 2+), E-cadherin(invasive ductal carcinoma+), cytokeratin (CK) 5/6(squamous carcinoma+), P40(squamous carcinoma+), cytokeratin (CK) 7(invasive ductal carcinoma+), pan-cytokeratin (CK-pan) (carcinoma+), vimentin(sarcoma+), smooth muscle actin (SMA) (sarcoma focal+). The patient, with a height of 160cm, weight of 68kg, and body surface area of 1.75m², recovered well postoperatively, and based on the condition and pathology results, was given 5 cycles of AC regimen adjuvant chemotherapy (doxorubicin 70mg per cycle, ifosfamide 4000mg per cycle, every 21 days). No radiotherapy was administered. After chemotherapy, the patient was lost to follow-up, and attempts to contact her or her relatives were unsuccessful.

## 3 Discussion

We report a case of carcinosarcoma, a malignant breast tumor comprising squamous cell carcinoma, invasive ductal carcinoma, and high-grade fibrosarcoma components. Carcinosarcoma, an aggressive form of metaplastic breast cancer (MpBC), represents less than 1% of all breast cancers (4). This cancer mainly affects postmenopausal women aged 49-61 years (5, 6). The tumor typically manifests as a rapidly growing mass, averaging 2.0cm to 5.5cm in diameter. Despite their large size, these tumors rarely involve axillary lymph nodes (6–8). Instead, early blood-borne metastasis to organs like the liver and lungs is more prevalent (9). Previous studies have shown that MpBC often presents benign imaging characteristics on mammography and ultrasound. Mammographic findings typically reveal a high- or iso-dense oval or irregular mass with narrow, indistinct, or ill-defined margins. On ultrasound, it frequently appears as a simple hypoechoic mass with similarly narrow or poorly defined borders (6). The patient first identified a lesion in her left breast at 63, and 5 years had passed by the time of her initial consultation. The presence of multiple tumor components resulted in a unique growth pattern, with the tumor rapidly expanding to nearly half the breast’s volume within two years before consultation. Imaging revealed the tumor’s expansive growth without evidence of axillary lymph node involvement. Ultrasound and mammography suggested a benign tumor, while pulmonary and abdominal CT scans found no metastatic lesions, rendering the imaging results nonspecific (5, 6, 8, 10). Consistent with previous study findings (6). This factor has impeded clinicians’ capacity for accurate carcinosarcoma diagnosis. Despite preoperative core needle biopsy, the lesion’s high heterogeneity (11) rendered the small sample insufficient for pathological diagnosis, complicating accurate preoperative assessment (9).

Regarding immunohistochemistry, previous studies have relatively consistently concluded that most tumors exhibit a triple-negative phenotype, with a minority being ER/PR-positive or HER2-positive (12–15). The study found no statistically significant association between hormone receptor status and survival outcomes (12, 16). Differences in HER2 status are also unlikely to contribute to variations in survival (15). However, one study involving 13 patients with MpBC found an association between hormone receptor expression and lymph node metastasis, as well as a correlation between HER2 expression and tumor histologic grade, tumor size, and lymph node metastasis (14). Given the rarity of MpBC—and the even lower prevalence of hormone receptor-positive or HER2-positive cases—it remains uncertain whether hormone receptor status and HER2 expression significantly impact prognosis. Larger clinical studies are needed in the future to validate these findings.

The absence of extensive clinical trials on MpBC means there are no definitive treatment guidelines (9). Thus, treatment decisions rely on clinical staging and the patient’s immunohistochemical phenotype at consultation. MpBC ‘s hallmark is the transformation of tumor epithelium into squamous and/or mesenchymal components. Treatment recommendations generally follow those for invasive breast cancer (8, 9). Most patients are triple-negative (7, 17), yet they respond less effectively to neoadjuvant chemotherapy than typical triple-negative cancers, showing a complete response rate of around 10% (6, 9, 18). Consequently, surgery plus adjuvant therapy is the preferred treatment (6). In this case, the Her-2 receptor was scored as 2+, but the patient declined further clarification of Her-2 gene status via FISH testing. Despite the undetermined Her-2 gene status, the postoperative treatment plan leans towards managing a triple-negative phenotype.

Due to the tumor’s large size, which disqualified the patient for breast-conserving surgery, the primary treatment option was mastectomy with axillary sentinel lymph node biopsy or dissection (9). Research indicates that MpBC patients undergoing postoperative adjuvant radiotherapy have a 30% lower mortality rate compared to those who do not receive radiation, highlighting the potential benefits of radiation therapy (6, 19). The selection of postoperative adjuvant chemotherapy regimens is guided by the status of estrogen and progesterone receptors, HER2 expression, and TNM staging. Studies suggest that squamous epithelial component cases benefit from platinum-based chemotherapy, while sarcomatous component cases respond well to anthracycline and cyclophosphamide-based regimens (5). Moreover, the presence of BRCA gene mutations in some patients indicates potential benefits from poly (ADP-ribose) polymerase inhibitor therapy (5).

The World Health Organization (WHO) classifies MpBC into six subtypes based on the mesenchymal and epithelial components of the tumor: (1) low-grade adenosquamous carcinoma, (2) fibromatosis-like metaplastic carcinoma, (3) squamous cell carcinoma, (4) spindle cell carcinoma, (5) metaplastic carcinoma with heterologous mesenchymal differentiation, and (6) mixed metaplastic carcinoma (6, 20). This case falls under the mixed metaplastic carcinoma subtype. Previous studies have found that fibromatosis-like metaplastic carcinoma and low-grade adenosquamous carcinoma are relatively sluggish. In contrast, other metaplastic variants tend to be aggressive, chemotherapy-resistant, and highly prone to metastasis (21, 22). Two large studies reported better survival rates for patients with metaplastic carcinoma exhibiting heterologous mesenchymal differentiation (23, 24). Regarding which subtype has the poorest survival rate, a series study involving 132 patients identified a lower survival rate in patients with metaplastic squamous cell carcinoma (23). Another study with 364 patients reported poorer clinical outcomes in those with spindle cell carcinoma (24). Additionally, some research suggests that patients with mixed metaplastic carcinoma may have lower survival rates than those with other subtypes (12, 20, 25). Due to the rarity of MpBC, large-scale clinical data are still needed to determine whether statistically significant prognostic differences exist between subtypes.

Our PubMed search for literature on metaplastic breast cancers with more than two histological types yielded only a single report meeting our criteria. We compared the characteristics of our case with the one found in the literature, as detailed in **Table 1**.

**Table 1:** Comparison of clinical manifestations, imaging findings, treatment, and prognosis between the two cases.

Feature	Chao Li et al. (26)	This Case
Admission Year	2018	2018
Gender	Female	Female
Age	77	68
Tumor Size	10.0cm×10.0cm	6.0cm × 6.0cm
Breast Affected	Right	Left
Location in Breast	Outer quadrant	Upper outer quadrant
Mammography Findings	Not performed	Slightly dense mass, no calcifications
Histological Type	Squamous cell carcinoma, invasive ductal carcinoma, and high-grade sarcoma	Squamous cell carcinoma (about 25%) + invasive ductal carcinoma (about 5%) + high-grade fibrosarcoma (about 70%)
Metastasis	Axillary lymph node, bone, lung	None observed
Surgery	Palliative mastectomy	Simple mastectomy and sentinel lymph node biopsy
Chemotherapy	Doxorubicin + Cyclophosphamide, Paclitaxel, Capecitabine	Doxorubicin + Ifosfamide
Radiotherapy	Not performed	Not performed
Prognosis	Lung metastasis reappeared 7 months post-surgery, treated with albumin-bound paclitaxel and carboplatin, alive at 11-month follow-up	Lost to follow-up

Literature indicates MpBC generally has a poor long-term prognosis (9–11), identifying surgical treatment and TNM staging as independent predictors of overall survival. Higher TNM stages correlate with lower overall survival rates, while surgical intervention improves these rates (18). Due to the loss of follow-up, the precise prognosis for our reported patient remains unknown; Chao Li et al. (26) described a malignant breast tumor case with three histological types and existing bone and lung metastases at diagnosis. The patient underwent a palliative mastectomy and survived for at least 11 months postoperatively. Our case, also featuring a tumor with three histological types, was diagnosed with the lesion confined to the breast, with no local lymph node or distant metastasis. Given the postoperative systemic treatment and lack of metastasis at diagnosis, we speculate our patient’s prognosis surpasses that in Chao Li et al.’s report. Despite the loss to follow-up, we surmise survival exceeded 11 months post-surgery.

We present a rare case of MpBC featuring three distinct tissue types, characterized by a large tumor with a propensity for skin invasion. Imaging studies provided nonspecific results, and accurate diagnosis depended on a comprehensive pathological examination of the tumor. Surgery is the primary treatment, and although prognosis is generally poor, early detection and treatment, alongside advancements in immunotherapy, can enhance both cure and survival rates.

## Data Availability

The original contributions presented in the study are included in the article/supplementary material. Further inquiries can be directed to the corresponding author.
